# Changes in Adult Alcohol Use and Consequences During the COVID-19 Pandemic in the US

**DOI:** 10.1001/jamanetworkopen.2020.22942

**Published:** 2020-09-29

**Authors:** Michael S. Pollard, Joan S. Tucker, Harold D. Green

**Affiliations:** 1 RAND Corporation, Santa Monica, California; 2 Indiana University School of Public Health, Bloomington, Indiana

## Abstract

This survey study examines individual-level changes in alcohol use in US adults and associated negative consequences, from before to during the coronavirus disease 2019 (COVID-19) pandemic.

## Introduction

As stay-at-home orders began in some US states as a mitigation strategy for coronavirus disease 2019 (COVID-19) transmission, Nielsen reported a 54% increase in national sales of alcohol for the week ending March 21, 2020, compared with 1 year before; online sales increased 262% from 2019.^1^ Three weeks later, the World Health Organization warned that alcohol use during the pandemic may potentially exacerbate health concerns and risk-taking behaviors.^2^ This study examines individual-level changes in alcohol use and consequences associated with alcohol use in US adults, as well as demographic disparities, from before to during the COVID-19 pandemic.

## Methods

In this survey study, data were collected using the RAND Corporation American Life Panel (ALP), a nationally representative, probability-sampled panel of 6000 participants age 18 years or more who speak English or Spanish; data are weighted to match a range of national demographic characteristics.^3^ Panel members provide informed consent annually online. All procedures were approved by the RAND Corporation Human Subjects Protection Committee. A sample of 2615 ALP members ages 30 to 80 years was invited to participate in the baseline survey (wave 1), which was closed after 6 weeks (April 29-June 9, 2019) with 1771 completions. Wave 2 data were collected from May 28 to June 16, 2020, several months after widespread implementation of COVID-19–associated social distancing. This study followed the American Association for Public Opinion Research (AAPOR) reporting guideline for survey studies.

The completion rate for the wave 2 survey was 58.9% of all wave 1 invitations. The ALP is composed of individuals recruited from multiple sources over more than 10 years, and a precise standardized response rate is difficult to compute. Based on survey completion rates of 56.6%, a prior estimate of the average ALP cumulative response rate is 9%.^4^

Comparisons before and during the COVID-19 pandemic were made on number of days of any alcohol use and heavy drinking (defined as 5 or more drinks for men and 4 or more drinks for women within a couple of hours), and average number of drinks consumed over the past 30 days. The 15-item Short Inventory of Problems^5^ assessed adverse consequences associated with alcohol use in the past 3 months (eg, “I have taken foolish risks when I have been drinking”). Comparisons were made overall, and across self-reported sex, age, and race/ethnicity. Significant changes were assessed based on whether the 95% CI around change from wave 1 to wave 2 included 0. Analyses include weights.

## Results

The current analytic sample includes 1540 adults (87.0%; mean [SD] age, 56.6 [13.5] years; 825 [53.6%] were in the age range of 30-59 years; and 883 [57.3%] were female) from the baseline survey who, approximately 1 year later, completed the wave 2 survey (Table 1). Frequency of alcohol consumption increased (1) overall, 0.74 days (95% CI, 0.33-1.15 days), representing an increase of 14% over the baseline of 5.48 days in 2019; (2) for women, 0.78 days (95% CI, 0.41-1.15 days), representing an increase of 17% over the 2019 baseline of 4.58 days; (3) for adults age 30 to 59 years, 0.93 days (95% CI, 0.36-1.51 days), an increase of 19%; and (4) for non-Hispanic White individuals, 0.66 days (95% CI, 0.14 to 1.17 days), an increase of 10% over the 2019 baseline of 6.46 days (Table 2). On average, alcohol was consumed 1 day more per month by 3 of 4 adults. For women, there was also a significant increase of 0.18 days of heavy drinking (95% CI, 0.04-0.32 days), from a 2019 baseline of 0.44 days, which represents an increase of 41% over baseline. This equates to an increase of 1 day for 1 in 5 women. For women there was an average increase in the Short Inventory of Problems scale of 0.09 (95% CI, 0.01-0.17 items), over the 2019 average baseline of 0.23, representing a 39% increase, which is indicative of increased alcohol-related problems independent of consumption level for nearly 1 in 10 women.

**Table 1.** Demographic Characteristics of Analytic Sample at 2019 Baseline, Unweighted (N = 1540)

Characteristic	No. (%)
Age group, y	
30-59	825 (53.6)
60-80	715 (46.4)
Mean (SD) age, y	56.6 (13.5)
Sex	
Female	883 (57.3)
Male	657 (42.7)
Race/ethnicity	
Non-Hispanic White	1099 (71.4)
Non-Hispanic Black	151 (9.8)
Hispanic	214 (13.9)
Other	76 (4.9)

**Table 2.** Estimates of Change in Alcohol Use and Associated Consequences From 2019 to 2020^a^

Health measure	Unit of measure (95% CI)								
	Overall	Men	Women	Age, y		Race/ethnicity			
				30-59	60-80	Non-Hispanic White	Non-Hispanic Black	Other	Hispanic
**Days consumed alcohol, past 30 d**									
Change from 2019	0.74 (0.33 to 1.15)	0.69 (−0.06 to 1.44)	0.78 (0.41 to 1.15)	0.93 (0.36 to 1.51)	0.37 (−0.11 to 0.84)	0.66 (0.14 to 1.17)	0.85 (−0.08 to 1.77)	0.94 (−0.38 to 2.26)	0.89 (−0.24 to 2.03)
Baseline days	5.48 (4.88 to 6.08)	6.45 (5.37 to 7.52)	4.58 (4.01 to 5.15)	4.98 (4.19 to 5.76)	6.41 (5.52 to 7.31)	6.46 (5.64 to 7.27)	3.13 (1.89 to 4.38)	4.11 (2.41 to 5.81)	3.91 (2.78 to 5.04)
**No. of drinks, past 30 d**									
Change from 2019	0.06 (−4.00 to 4.13)	1.00 (−6.13 to 8.14)	−0.81 (−5.04 to 3.43)	2.82 (−1.11 to 6.75)	−5.09 (−14.09 to 3.90)	0.16 (−4.57 to 4.90)	5.75 (−4.96 to 16.47)	5.52 (−3.48 to 14.53)	−5.95 (−19.15 to 7.25)
Baseline drinks, past 30 d	18.47 (14.01 to 22.84)	22.08 (15.04 to 29.12)	15.13 (9.82 to 20.45)	16.38 (11.83 to 20.93)	22.39 (13.20 to 31.58)	18.57 (13.68 to 23.45)	18.31 (0.73 to 35.89)	9.38 (5.53 to 13.24)	22.5 (8.80 to 36.21)
**Heavy drinking days, past 30 d**^**b**^									
Change from 2019	0.13 (−0.09 to 0.34)	0.07 (−0.36 to 0.49)	0.18 (0.04 to 0.32)	0.23 (−0.05 to 0.51)	−0.07 (−0.39 to 0.25)	0.16 (−0.03 to 0.35)	0.27 (−0.53 to 1.06)	−0.49 (−1.41 to 0.44)	0.14 (−0.67 to 0.96)
Baseline heavy drinking days, past 30 d	0.69 (0.46 to 0.92)	0.95 (0.55 to 1.36)	0.44 (0.22 to 0.66)	0.79 (0.48 to 1.10)	0.50 (0.19 to 0.80)	0.44 (0.30 to 0.58)	1.02 (−0.03 to 2.07)	1.37 (−0.58 to 0.11)	1.22 (0.36 to 2.08)
**SIP scale, past 3 mo**									
Change from 2019	0.09 (−0.02 to 0.21)	0.10 (−0.13 to 0.33)	0.09 (0.01 to 0.17)	0.13 (−0.05 to 0.31)	0.03 (−0.04 to 0.10)	0.05 (−0.04 to 0.14)	−0.06 (−0.20 to 0.08)	−0.24 (−0.58 to 0.11)	0.48 (−0.07 to 1.04)
Baseline SIP, past 3 mo	0.30 (0.22 to 0.37)	0.37 (0.25 to 0.50)	0.23 (0.15 to 0.30)	0.38 (0.27 to 0.48)	0.15 (0.07 to 0.22)	0.31 (−0.22 to 0.41)	0.29 (0.13 to 0.45)	0.32 (−0.03 to 0.67)	0.22 (0.06 to 0.37)
No.^c^	1520 to 1529	648 to 652	868 to 877	812 to 820	701 to 710	1085 to 1090	147	76	211 to 213

Abbreviation: SIP, Short Inventory of Problems.

^a^ Change was measured between baseline (April 29-June 9, 2019) and wave 2 (May 28-June 16, 2020).

^b^ Heavy drinking constitutes 5 or more drinks for men and 4 or more drink for women within a couple of hours.

^c^ Sample size ranges are due to item nonresponse.

## Discussion

These data provide evidence of changes in alcohol use and associated consequences during the COVID-19 pandemic. In addition to a range of negative physical health associations, excessive alcohol use may lead to or worsen existing mental health problems, such as anxiety or depression,^6^ which may themselves be increasing during COVID-19. The population level changes for women, younger, and non-Hispanic White individuals highlight that health systems may need to educate consumers through print or online media about increased alcohol use during the pandemic and identify factors associated with susceptibility and resilience to the impacts of COVID-19.

Study limitations include that measures are self-reports, which may be subject to social desirability bias. Additionally, not all baseline respondents completed wave 2, although nonrespondents did not significantly differ from completers on any of the outcome measures at baseline. Nonetheless, these results suggest that examination of whether increases in alcohol use persist as the pandemic continues and whether psychological and physical well-being are subsequently affected may be warranted.
